# Analysis of Factors Associated With Interstitial Lung Disease in Treatment‐Naïve Rheumatoid Arthritis Patients and Development of a Classification Nomogram

**DOI:** 10.1002/iid3.70571

**Published:** 2026-10-29

**Authors:** Li Lu, Yongkang Chen, Ting Zhang, Lina Cao, Hua Ma, Yuanyuan Song, Dongmei Zhou

**Affiliations:** ^1^ Department of Rheumatology and Immunology The Affiliated Hospital of Xuzhou Medical University Xuzhou China

**Keywords:** cytokines, IL‐22, interstitial lung disease, nomogram, rheumatoid arthritis, TNF‐α

## Abstract

**Background:**

Interstitial lung disease (ILD) is a serious extra‐articular manifestation of rheumatoid arthritis (RA) and contributes substantially to morbidity and mortality. Early identification of RA‐associated ILD (RA‐ILD) remains challenging, and reliable biomarkers for risk stratification are lacking.

**Objective:**

This study aimed to identify cytokine‐related factors associated with RA‐ILD and to develop an exploratory classification model for assessing the presence of ILD in patients with newly diagnosed, treatment‐naïve RA.

**Methods:**

A total of 66 newly diagnosed, treatment‐naïve RA patients were enrolled, including individuals with and without ILD confirmed by high‐resolution computed tomography (HRCT). All patients were disease‐modifying antirheumatic drug (DMARD)‐naïve and had not received glucocorticoids or biologics prior to enrollment. Plasma IL‐22 concentration was measured by ELISA; other cytokines (IL‐2, IL‐4, IL‐6, IL‐10, IL‐12p70, IL‐17A, IFN‐γ, and TNF‐α) were quantified using a multiplex bead‐based immunoassay. Clinical characteristics, laboratory parameters, and radiological findings were collected. Feature selection was performed using least absolute shrinkage and selection operator (LASSO) regression and Boruta algorithms, followed by multivariable logistic regression to identify independent factors associated with RA‐ILD. A nomogram was constructed based on selected predictors, and its performance was evaluated using receiver operating characteristic analysis and cross‐validation.

**Results:**

Patients with RA‐ILD exhibited distinct cytokine profiles compared with those without ILD. Multivariable analysis identified age, smoking, lower mean corpuscular hemoglobin concentration (MCHC), higher plasma TNF‐α levels and lower plasma IL‐22 levels as independent factors associated with RA‐ILD. The nomogram exhibited good discriminative performance, with a fivefold cross‐validated AUC of 0.851 (95% CI: 0.755–0.947); the apparent AUC in the full sample was 0.87. Internal validation indicated acceptable model stability.

**Conclusion:**

Age, smoking, MCHC, higher plasma TNF‐α levels and lower plasma IL‐22 levels were associated with the presence of ILD in newly diagnosed, treatment‐naïve RA patients. The proposed cytokine‐based nomogram may serve as a hypothesis‐generating tool for early identification of RA‐ILD. Further validation in larger, independent cohorts is warranted before clinical application.

## 1 Introduction

Rheumatoid arthritis (RA) is a chronic, progressive systemic autoimmune disorder characterized by synovial inflammation, joint destruction, and various extra‐articular manifestations [1, 2, 3]. Among these, rheumatoid arthritis‐associated interstitial lung disease (RA‐ILD) represents a significant complication involving progressive pulmonary fibrosis that substantially contributes to mortality in RA patients [4, 5, 6, 7]. Despite its clinical significance, the pathogenesis of RA‐ILD remains incompletely understood, necessitating further investigation into potential biomarkers and therapeutic targets. Rheumatoid arthritis is a systemic autoimmune disease driven by immune dysregulation, including autoantibody production, T‐cell imbalance, and cytokine‐mediated inflammation. In a subset of patients, this systemic inflammation extends to the lungs, where persistent immune activation leads to fibroblast proliferation and progressive fibrosis, culminating in interstitial lung disease (ILD). RA‐ILD is one of the most serious extra‐articular manifestations and accounts for approximately 10‐20% of mortality in RA patients. However, the precise immunological mechanisms that predispose certain patients to develop pulmonary fibrosis remain poorly understood, and reliable serum biomarkers for early RA‐ILD detection are urgently needed.

Previous studies have demonstrated that IL‐22 [8] expression may be reduced in RA‐ILD patients, with levels potentially inversely correlating with the severity of pulmonary involvement [9]. Experimental validation using murine models of pulmonary fibrosis has suggested a protective role of IL‐22 against fibrotic changes [10]. Mechanistic insights revealed that IL‐22 may decrease Th17 cell proportion and reduce IL‐17A mRNA levels [11], suggesting that IL‐22 may exert anti‐fibrotic effects partly through suppression of the IL‐17 pathway.

We therefore aimed to analyze cytokine profiles in peripheral blood samples from RA and RA‐ILD patients, focusing on IL‐22 and IL‐17A. Additionally, we evaluated other cytokines including IL‐2, IL‐4, IL‐6, IL‐10, IL‐12p70, IFN‐γ, and TNF‐α [12, 13, 14, 15, 16, 17] to identify a comprehensive profile associated with RA‐ILD. Accordingly, we developed a nomogram to identify factors associated with RA‐ILD, providing a tool that may assist clinical assessment and contribute to understanding of RA‐ILD pathophysiology.

## 2 Methods

### 2.1 Study Design and Population

This retrospective case‐cohort study included 66 adult patients with newly diagnosed, treatment‐naïve rheumatoid arthritis (RA) consecutively recruited from the Department of Rheumatology and Immunology at the Affiliated Hospital of Xuzhou Medical University between October 2021 and October 2022. All patients were DMARD‐naïve and had not received glucocorticoids or biologics prior to enrollment. RA was diagnosed according to the 2010 classification criteria jointly established by the American College of Rheumatology (ACR) and the European League Against Rheumatism (EULAR) [18]. Patients with a history of malignancy or other concomitant autoimmune diseases were excluded. All participants underwent high‐resolution computed tomography (HRCT) of the chest for interstitial lung disease (ILD) screening at enrollment. The diagnosis of ILD was made in accordance with the official guidelines of the American Thoracic Society (ATS) and European Respiratory Society (ERS) [19]. Based on HRCT findings, patients were stratified into two groups: those with RA‐associated ILD (RA‐ILD, *n* = 33) and those without ILD (RA‐non‐ILD, *n* = 33). The 33 RA patients without ILD were consecutively selected from the same source population during the same enrollment period (October 2021–October 2022). The 1∶1 balanced design was intended to ensure comparable sample sizes between the two groups to enhance the stability of model parameter estimation, rather than to simulate real‐world prevalence. No matching or randomization was performed in the selection of patients without ILD. The study protocol was approved by the Ethics Committee of the Affiliated Hospital of Xuzhou Medical University (approval number: XYFY2022‐KL104‐01), and written informed consent was obtained from all participants.

### 2.2 Sample Collection and Processing

Fasting peripheral venous blood (3 mL) was collected from each participant into EDTA‐containing tubes. Samples were gently mixed and stored temporarily at 4°C before being centrifuged at 2000 rpm for 15 min (rotor radius: 20 cm; approximately 90 × g). Plasma supernatants were carefully aliquoted and stored at ‐80°C until analysis, with freeze‐thaw cycles avoided.

### 2.3 Cytokine Measurements

Levels of IL‐22 were measured with ELISA kits for human IL‐22 according to the manufacturer's protocols (model: RT‐6100, Rayto Life Sciences Co. Ltd. China). Concentrations of IL‐2, IL‐4, IL‐6, IL‐10, IL‐12p70, IL‐17A, IFN‐γ, and TNF‐α were conducted on a Luminex MAGPIX system, which is a liquid suspension chip‐based detection instrument (the kit was obtained from Nanjing Aituo Life Science Technology Co. Ltd., and the reagent specification is 96 tests per kit). All assays were performed strictly according to the manufacturers' instructions.

### 2.4 HRCT Scoring of Pulmonary Fibrosis

The extent of pulmonary fibrosis was assessed on HRCT images at three axial levels: the carina, the aortic arch, and 1 cm above the diaphragm. At each level, the percentage of lung parenchyma affected by fibrotic changes was estimated. A semi‐quantitative scoring system was applied based on the total involvement across all three levels: 0% (score 0), 1%–25% (score 1), 26%–50% (score 2), 51%–75% (score 3), and > 75% (score 4). The resulting HRCT fibrosis score ranged from 0 to 4. Two experienced radiologists, blinded to clinical data, independently reviewed all scans; discrepancies were resolved through consensus discussion.

### 2.5 Statistical Analysis

Statistical analyses were performed using SPSS version 27.0 (IBM Corp., Armonk, NY, USA) and R software version 4.3.1 (Vienna, Austria). Categorical variables were presented as frequencies and percentages [n (%)], while continuous variables were expressed as mean ± standard deviation (SD) for normally distributed data or median with interquartile range [IQR] for data that violated normality assumptions. Group comparisons were conducted using the independent samples t‐test for normally distributed variables and the Wilcoxon rank‐sum test for non‐normal distributions. Univariate logistic regression was used to identify factors associated with RA‐ILD. To select the most parsimonious set of variables and avoid overfitting, least absolute shrinkage and selection operator (LASSO) regression and Boruta feature selection were applied. Variables selected by LASSO, along with smoking based on its established clinical relevance, were included in the final model. A nomogram was developed based on this model to estimate the probability of having RA‐ILD. Model performance was evaluated using fivefold cross‐validation. Discrimination was assessed by the area under the receiver‐operating characteristic curve (AUC), calibration was examined via a calibration plot, and clinical utility was evaluated using decision curve analysis (DCA). A two‐sided *p* < 0.05 was considered statistically significant. To account for potential confounding by age and smoking history, multivariable linear regression models were fitted with each cytokine as the dependent variable and RA‐ILD status as the main independent variable, adjusting for age and smoking history. IL‐22 was analyzed on the original scale, whereas non‐normally distributed cytokines were analyzed after natural logarithmic transformation. Partial Spearman correlation analyses were additionally performed to assess correlations among cytokines and between cytokines and clinical parameters, controlling for age and smoking history. Given the exploratory nature of this study, no formal adjustment was applied for multiple comparisons across the nine cytokines and multiple clinical variables tested. Findings should therefore be interpreted conservatively, and the identified associations warrant confirmation in independent cohorts.

## 3 Results

### 3.1 Demographic and Clinical Characteristics

Patients in the RA‐ILD group were significantly older (mean age: 66.15 ± 10.40 years) and had a higher prevalence of smoking (27.3%) compared to those in the RA‐non‐ILD group (mean age: 54.39 ± 11.06 years; smoking: 6.1%; both *p* < 0.05). No significant differences were observed between the two groups in terms of sex distribution, prevalence of hypertension, or coronary heart disease (*p* > 0.05 for all). The RA‐ILD group exhibited higher Disease Activity Score 28 (DAS28), swollen joint count (SJC), and tender joint count (TJC), as well as lower mean corpuscular hemoglobin concentration (MCHC), all with *p* < 0.05. Detailed demographic and clinical characteristics are summarized in Table 1.

**Table 1:** Demographic and clinical characteristics.

Characteristics	Overall (*N* = 66)	RA‐non‐ILD (*N* = 33)	RA‐ILD (*N* = 33)	*p* value
gender = Female (%)	54 (81.8)	28 (84.8)	26 (78.8)	0.75
Age (years, mean ± SD)	60.27 (12.19)	54.39 (11.06)	66.15 (10.40)	< 0.001
Smoking history n (%)	11 (16.7)	2 (6.1)	9 (27.3)	0.048
Hypertension *n* (%)	9 (13.6)	5 (15.2)	4 (12.1)	1
CHD *n* (%)	10 (15.2)	5 (15.2)	5 (15.2)	1
SJC (mean ± SD)	9.76 (8.02)	7.36 (7.05)	12.15 (8.32)	0.014
TJC (mean ± SD)	10.65 (8.26)	8.12 (6.62)	13.18 (9.04)	0.012
DAS28 (mean ± SD)	4.93 (1.41)	4.50 (1.24)	5.36 (1.46)	0.013
HRCT (%)				< 0.001
0	33 (50.0)	33 (100.0)	0 (0.0)	
1	5 (7.6)	0 (0.0)	5 (15.2)	
2	14 (21.2)	0 (0.0)	14 (42.4)	
3	10 (15.2)	0 (0.0)	10 (30.3)	
4	4 (6.1)	0 (0.0)	4 (12.1)	
ESR (mm/h, median [IQR])	40.00 [23.25, 60.75]	33.00 [23.00, 62.00]	43.00 [25.00, 59.00]	0.603
CRP (mg/dL, mean (SD))	25.37 (29.42)	23.00 (26.48)	27.73 (32.34)	0.518
MCHC (g/L, mean (SD))	317.80 (12.26)	321.96 (13.83)	313.64 (8.83)	0.005

Abbreviations: CHD, coronary heart disease; CRP, C‐reactive protein; DAS28, disease activity score; ESR, erythrocyte sedimentation rate; HRCT, high‐resolution computed tomography; MCHC, mean corpuscular hemoglobin concentration; RA‐ILD, rheumatoid arthritis‐associated interstitial lung disease; RA‐NILD, rheumatoid arthritis without interstitial lung disease; SJC, swollen joint count; TJC, tender joint count.

### 3.2 Plasma Cytokine Levels

Plasma IL‐22 levels were significantly lower in the RA‐ILD group (205.36 ± 44.81 pg/mL) than in the RA‐non‐ILD group (246.92 ± 41.53 pg/mL; *p* < 0.001). TNF‐α levels were significantly higher in the RA‐ILD group (median [IQR]: 2.43 [1.99, 5.56] pg/mL) compared to the RA‐non‐ILD group (1.65 [1.15, 2.91] pg/mL; *p* = 0.012). No significant between‐group differences were observed for IL‐17, IL‐2, IL‐4, IL‐6, IL‐10, IL‐12p70, or IFN‐γ (*p* > 0.05 for all). Cytokine concentrations are presented in Table 2 and Figure 1. Because IL‐22 concentrations were substantially higher than those of the other cytokines, IL‐22 is shown separately in Supporting Information S1: Figure S1. After adjustment for age and smoking history, IL‐22 remained significantly lower and TNF‐α remained significantly higher in the RA‐ILD group (Supporting Information S1: Table S1).

**Table 2:** Comparisons of cytokine levels between RA‐ILD and RA‐non‐ILD group.

Cytokines	Overall (*N* = 66)	RA‐ILD (*N* = 33)	RA‐non‐ILD (*N* = 33)	*p* value
IL_22 (pg/mL, mean ± SD)	226.14 (47.71)	205.36 (44.81)	246.92 (41.53)	< 0.001
IL‐17 (pg/mL, (median [IQR]))	2.80 [1.77, 6.94]	3.46 [1.78, 8.70]	1.94 [1.69, 5.72]	0.323
IL‐2 (pg/mL, (median [IQR]))	8.21 [2.22, 55.11]	9.62 [2.21, 58.06]	4.66 [2.24, 34.69]	0.547
IL‐6 (pg/mL, (median [IQR]))	8.84 [3.13, 17.45]	9.36 [4.89, 19.34]	8.28 [2.82, 16.58]	0.369
IL‐4 (pg/mL, (median [IQR]))	2.72 [1.35, 10.09]	4.15 [1.71, 11.04]	1.83 [1.22, 6.05]	0.078
IL‐10 (pg/mL, (median [IQR]))	2.12 [1.33, 2.90]	2.36 [1.63, 4.32]	1.78 [0.89, 2.65]	0.130
IL‐12P70 (pg/mL, (median [IQR]))	2.38 [1.76, 4.28]	3.31 [2.07, 5.13]	2.22 [1.60, 3.22]	0.094
IFN‐γ (pg/mL, (median [IQR]))	4.44 [1.66, 8.78]	4.58 [1.79, 12.34]	4.22 [1.40, 6.79]	0.359
TNF‐α (pg/mL, (median [IQR]))	2.26 [1.47, 3.68]	2.43 [1.99, 5.56]	1.65 [1.15, 2.91]	0.012

*Note:* Continuous variables were expressed as mean (SD) for normally distributed data and median (IQR) for non‐normally distributed data. *p* value obtained after comparison between RA‐ILD patients and RA patients without ILD.

**Figure 1:**
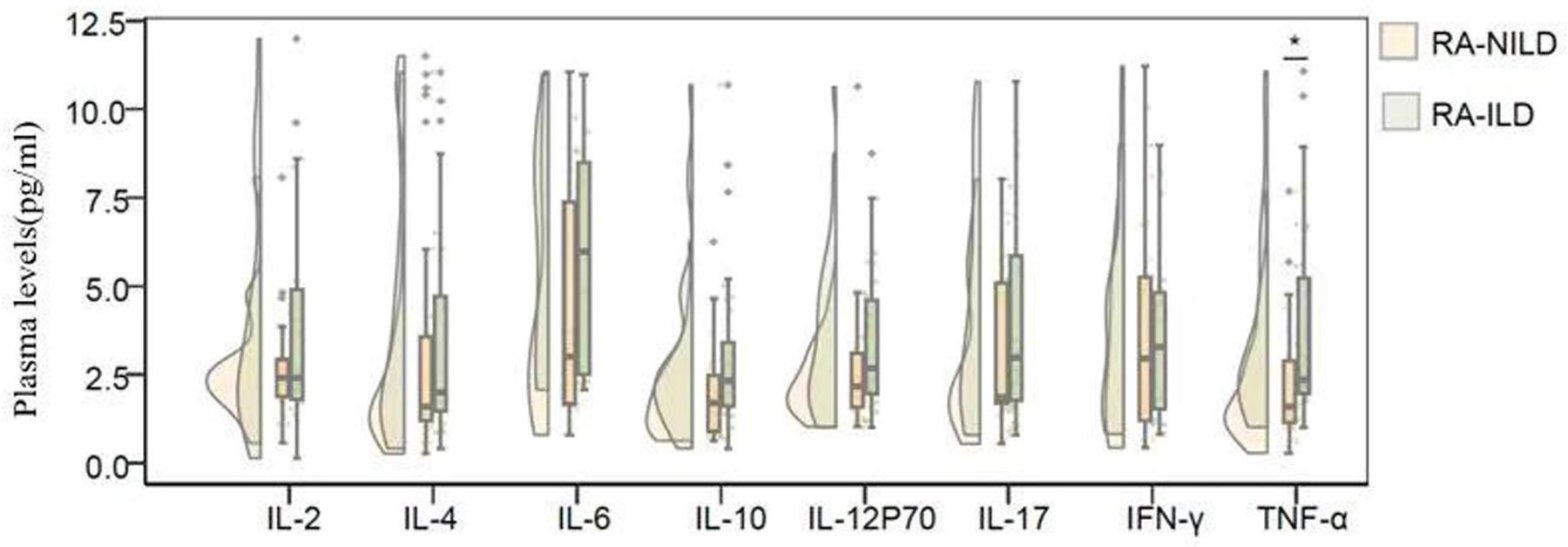
Comparisons of cytokine levels between RA‐ILD and RA‐non‐ILD group.

### 3.3 Correlation Analyses

Moderate to strong positive correlations were observed among several cytokines, particularly IL‐6, IL‐10, IL‐12p70, IFN‐γ, and TNF‐α. Notably, IL‐6, IL‐10, and TNF‐α showed significant positive correlations with clinical disease activity measures (DAS28, SJC, and TJC). Additionally, TNF‐α was positively correlated with HRCT fibrosis score, whereas IL‐22 was negatively correlated with HRCT fibrosis score. The full correlation network is shown in Figure 2. Partial Spearman correlation analyses adjusting for age and smoking history confirmed these associations (Supporting Information S1: Table S2).

**Figure 2:**
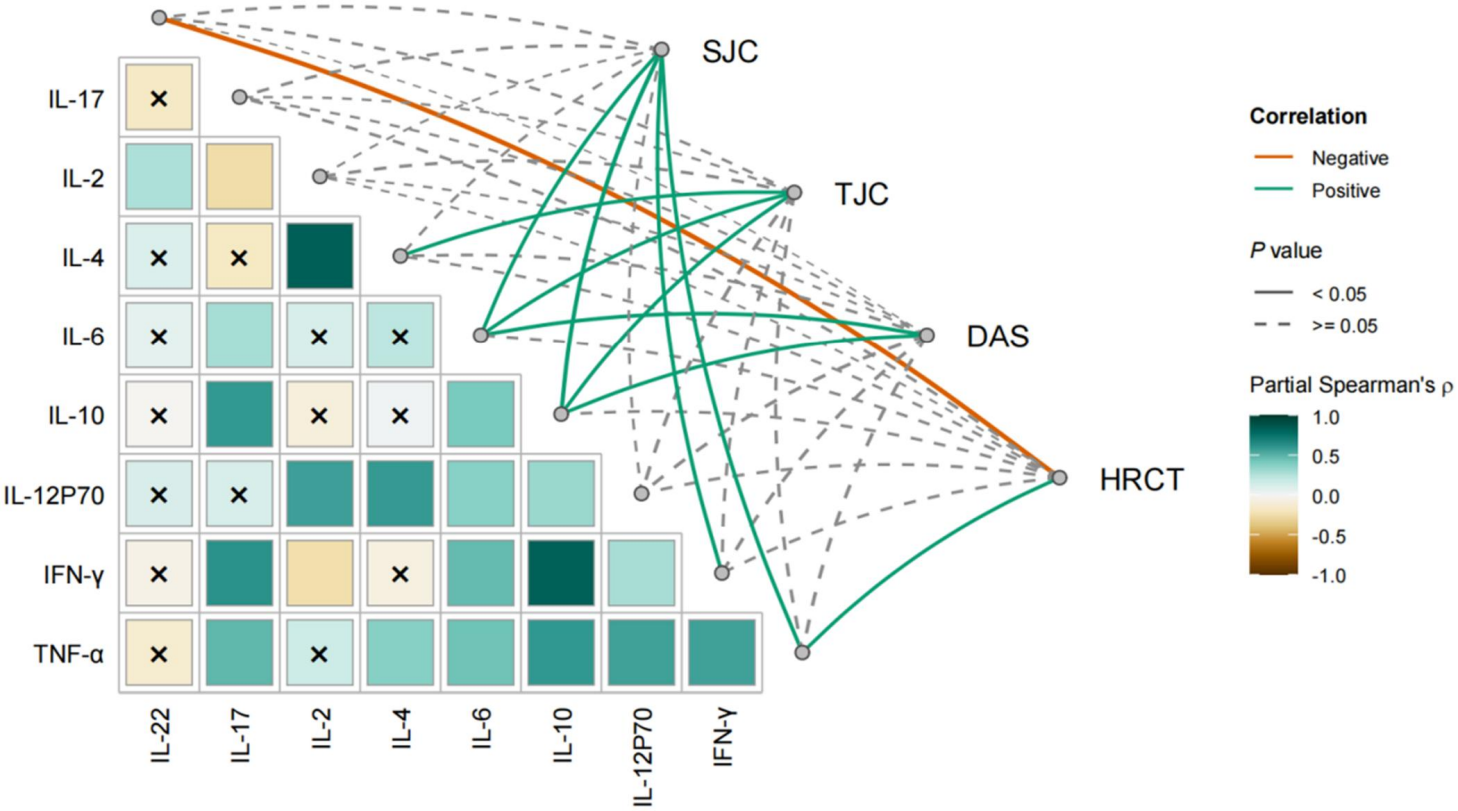
Integrated correlation network between immune cytokine profiles and disease‐related clinical parameters.

### 3.4 Diagnostic Performance of Biomarkers and Predictive Model

Univariable logistic regression showed that age, smoking status, IL‐22, MCV antibody, DAS28, MCHC, SJC, TJC, and TNF‐α were significantly associated with RA‐ILD (*p* < 0.05; Table 3). After applying LASSO and Boruta feature selection methods, five variables—age, MCHC, IL‐22, natural log‐transformed TNF‐α and smoking—were consistently selected for inclusion in the final model (Table 4). A nomogram based on these five factors (age, MCHC, IL‐22, ln_TNF‐α, and smoking) demonstrated good discriminative ability, with a fivefold cross‐validated area under the ROC curve (AUC) of 0.851 (95% CI: 0.755‐0.947; Figure 3). A sensitivity analysis excluding smoking yielded a cross‐validated AUC of 0.841 (Supporting Information S1: Figure S2), supporting the robustness of the final model. The nomogram incorporating age, MCHC, IL‐22, ln_TNF‐α, and smoking is illustrated in Figure 4. The calibration plot demonstrated good agreement between predicted and observed probabilities of RA‐ILD (Figure 5). Decision curve analysis showed that the nomogram provided greater net clinical benefit than either “treat‐all” or “treat‐none” strategies across a wide range of threshold probabilities (approximately 0.05 to 0.70; Figure 6).

**Table 3:** Univariable logistic regression.

Characteristics	OR (95% CI)	*p* value
Gender	0.66 (0.18, 2.34)	0.525
Age	1.10 (1.05, 1.17)	0.000
SJC	1.08 (1.02, 1.16)	0.017
TJC	1.08 (1.02, 1.16)	0.015
DAS	1.60 (1.11, 2.42)	0.017
Smoking	5.81 (1.34, 40.52)	0.033
Hypertension	0.77 (0.18, 3.21)	0.720
CHD	1.00 (0.25, 3.97)	1.000
ESR	1.00 (0.98, 1.02)	0.903
ln_CRP	1.17 (0.77, 1.81)	0.454
MCHC	0.93 (0.87, 0.97)	0.009
IL‐22	0.98 (0.96, 0.99)	0.001
ln_IL‐2	1.08 (0.83, 1.41)	0.583
ln_IL‐4	1.44 (1.01, 2.15)	0.055
ln_IL‐6	1.17 (0.79, 1.75)	0.433
ln_IL‐10	1.29 (0.78, 2.27)	0.338
ln_IL‐12P70	1.63 (0.94, 3.23)	0.118
ln_IL‐17	1.17 (0.73, 1.92)	0.515
ln_IFN‐γ	1.28 (0.90, 1.89)	0.184
ln_TNF‐α	2.30 (1.21, 4.97)	0.020

*Note:* Natural logarithm transformation was applied to CRP, TNF‐α, IL‐2, IL‐4, IL‐6, IL‐10, IL‐12p70, IL‐17, and IFN‐γ due to non‐normal distributions.

**Table 4:** Variables selected by univariable analysis, LASSO and Boruta.

Variables	Univariable logistic regression	LASSO	Boruta
Gender			
Age	√	√	√
SJC	√		
TJC	√	√	
DAS	√		√
Smoking	√	√	
Hypertension			
CHD			
ESR			
ln_CRP			
MCHC	√	√	√
IL_22	√	√	√
ln_IL_2			
ln_IL_4			
ln_IL_6			
ln_IL_10			
ln_IL_12P70			
ln_IL_17			
ln_IFN_γ			
ln_TNF_α	√	√	√

*Note:* Age, MCHC, IL‐22, ln_TNF_α and smoking were included in the final model for nomogram construction.

**Figure 3:**
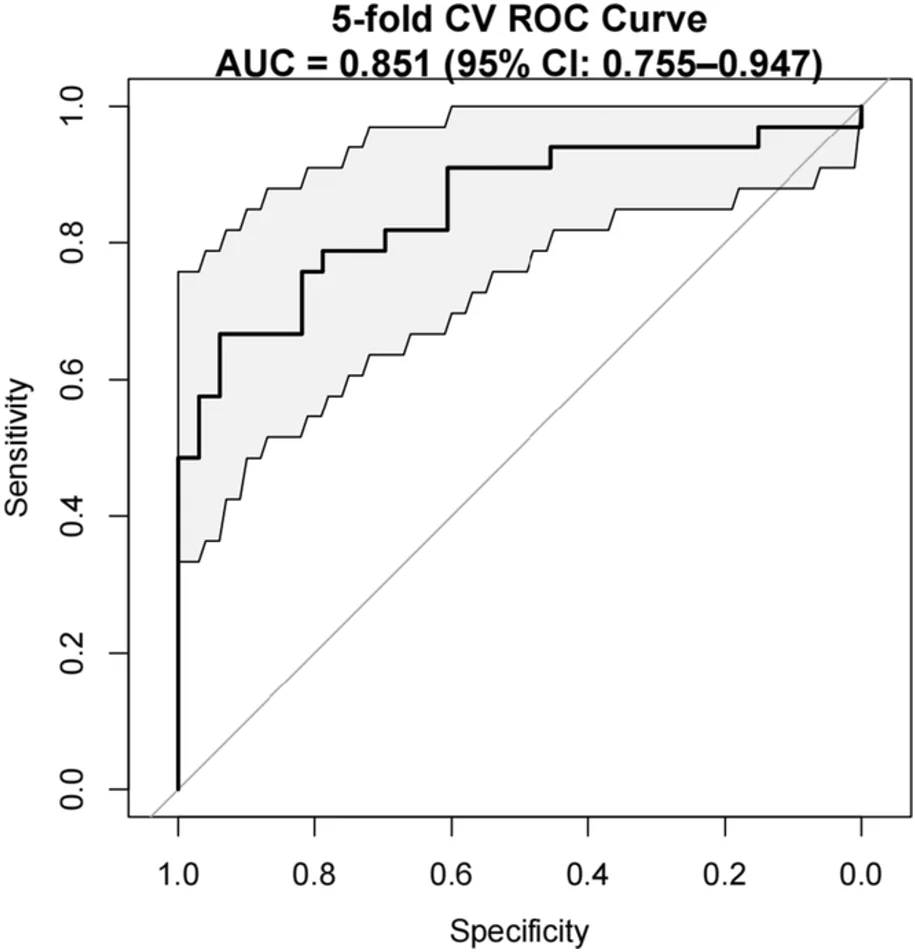
The discriminative performance of the logistic regression model was evaluated using fivefold cross‐validation. The cross‐validated ROC curve showed good predictive accuracy, with an AUC of 0.851 (95% CI: 0.755–0.947). The shaded region represents the 95% confidence interval of the cross‐validated ROC estimate, indicating stable performance across folds.

**Figure 4:**
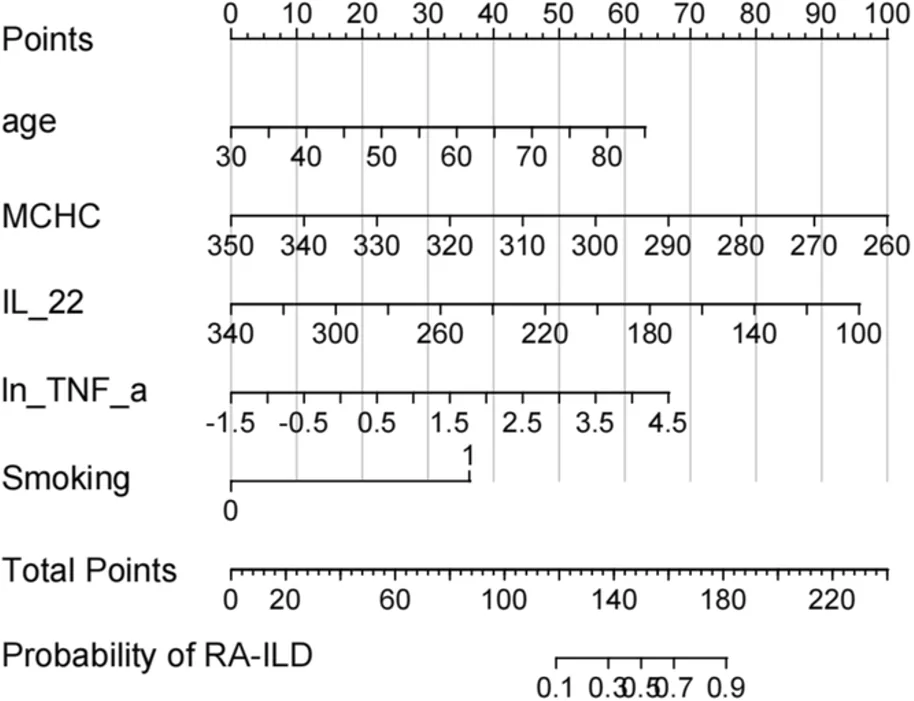
A nomogram was constructed based on age, MCHC, IL‐22, ln(TNF‐α), and smoking to estimate the individual probability of RA‐ILD. Each factor is assigned a point value according to its contribution to the model, and the sum of these points corresponds to the predicted risk of RA‐ILD at the bottom scale. Higher age, lower MCHC and IL‐22, elevated TNF‐α, and smoking contribute to an increased total point score, thereby indicating a higher probability of having RA‐ILD.

**Figure 5:**
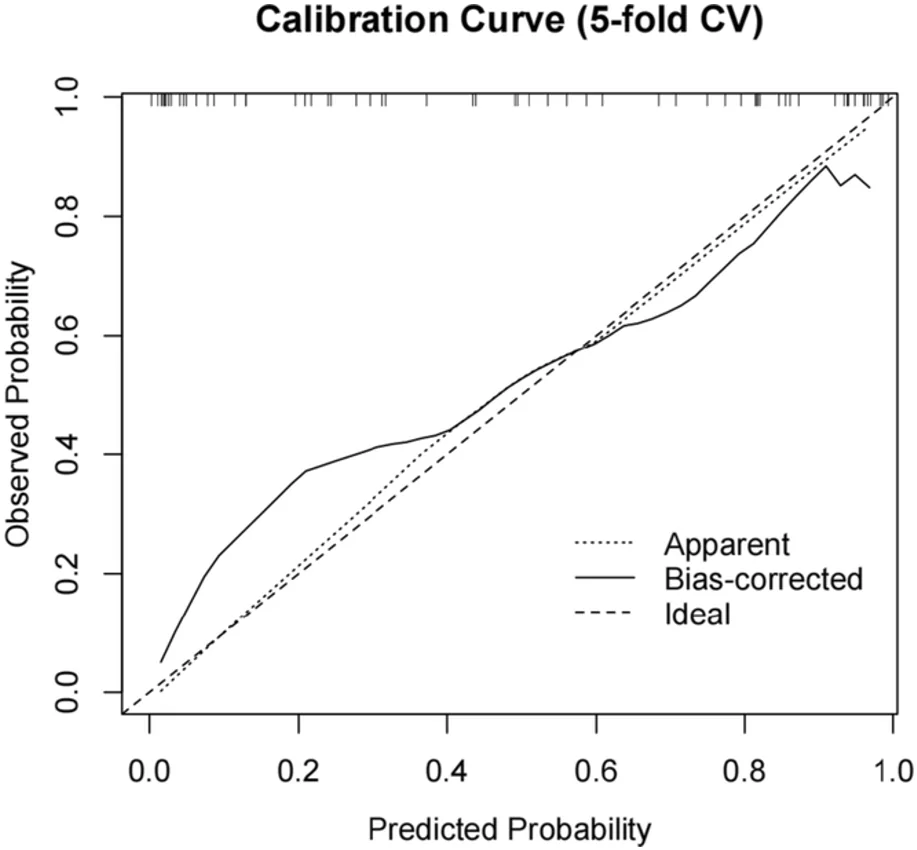
The fivefold cross‐validated calibration curve demonstrated acceptable agreement between predicted and observed probabilities of RA‐ILD.

**Figure 6:**
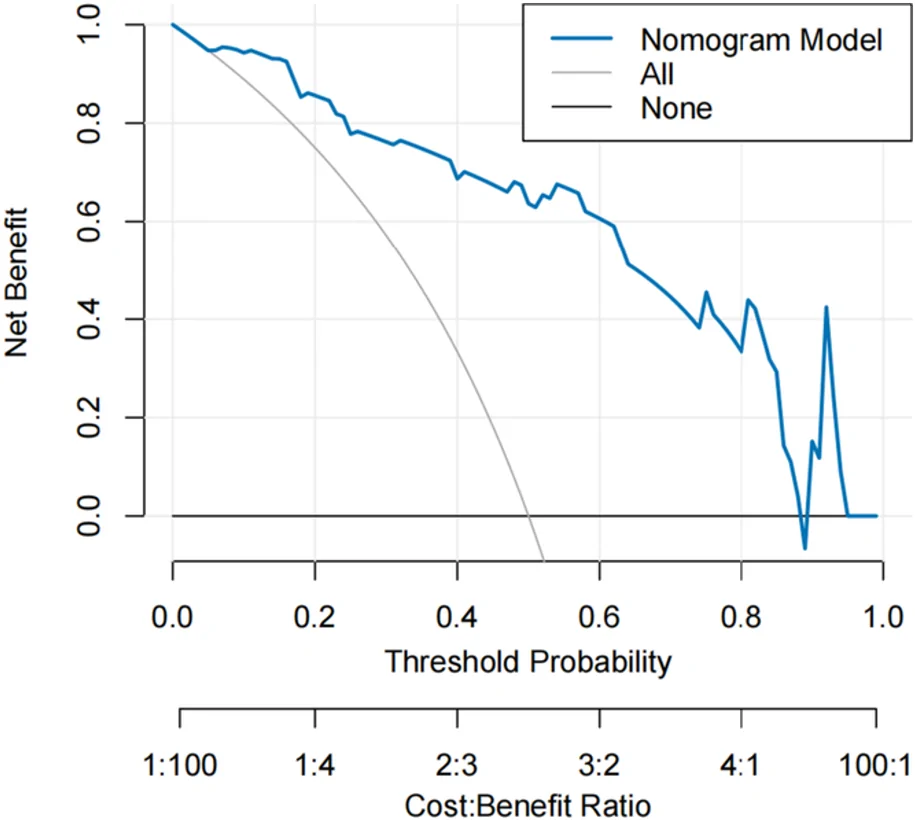
The decision curve analysis indicated that the nomogram model provided a higher net benefit than both the “treat‐all” and “treat‐none” strategies across a wide range of threshold probabilities (approximately 0.05–0.70). This suggests that using the model to guide clinical decisions would result in more benefit than treating all patients or treating none, particularly within the clinically relevant threshold range. At very high threshold probabilities (> 0.7), the net benefit of the model declined, indicating limited added value when clinicians require a very high predicted risk to justify intervention.

## 4 Discussion

In this study, we observed that plasma IL‐22 levels were significantly lower and TNF‐α levels significantly higher in RA patients with interstitial lung disease (RA‐ILD) compared to those without ILD (RA‐non‐ILD). These findings suggest that IL‐22 may be inversely associated with HRCT‐based fibrosis severity, whereas TNF‐α may show a positive association with both radiological extent and clinical disease activity. Using age, MCHC, IL‐22, TNF‐α and smoking as predictor variables, we developed a nomogram that demonstrated good discriminative performance (cross‐validated AUC = 0.851) for identifying RA‐ILD risk. It is important to note that our nomogram is designed for classifying existing RA‐ILD rather than predicting future onset. As a cross‐sectional study, our model identifies concurrent disease; prospective validation is required to assess its predictive utility for incident RA‐ILD.

These findings extend prior preclinical evidence suggesting that IL‐22 may play a protective role in pulmonary fibrosis [9, 11]. In murine models, IL‐22 deficiency appeared to exacerbate bleomycin‐induced lung inflammation and fibrosis [10], while exogenous IL‐22 administration appeared to attenuate these changes, possibly through suppression of Th17 cells and IL‐17A expression [11]. However, in the present clinical cohort, circulating IL‐17A levels did not differ significantly between RA‐ILD and RA‐non‐ILD groups—a discrepancy that may reflect compartmentalization of immune responses (e.g., local vs. systemic) or limited statistical power due to the modest sample size. This highlights the difficulty in translating mechanistic findings from animal models to human disease.

The elevated TNF‐α levels in RA‐ILD are consistent with its established pro‐inflammatory and pro‐fibrotic functions [20]. Importantly, TNF‐α can amplify inflammatory cascades by inducing other cytokines and has been implicated in alveolar epithelial cell apoptosis and impaired tissue repair [21, 22] —processes central to the pathogenesis of pulmonary fibrosis [23, 24]. The positive association between TNF‐α and both HRCT fibrosis score and disease activity indices (DAS28, SJC, TJC) further supports its potential role in linking systemic autoimmunity to lung involvement in RA [25].

Regarding MCHC as a predictor, we acknowledge that its association with RA‐ILD is not immediately obvious and that few reports have directly examined this relationship. However, low MCHC levels have been reported as a valuable biomarker for assessing the prognosis of pulmonary diseases, such as acute exacerbations of chronic obstructive pulmonary disease. We hypothesize that chronic systemic inflammation in RA may subtly affect erythrocyte parameters, and that MCHC may serve as a proxy for systemic inflammatory burden rather than a direct mediator of pulmonary fibrosis. Additionally, low MCHC may reflect impaired iron utilization or anemia of chronic disease, both of which are common in RA and may be more pronounced in patients with more severe systemic involvement, including ILD. Nevertheless, we emphasize that this association is exploratory and requires further mechanistic and validation studies.

The nomogram [26, 27] integrating clinical and cytokine variables offers a practical tool for risk stratification in clinical settings. Although smoking was not selected by Boruta, we included it in the final model based on its well‐established clinical relevance to RA‐ILD and the observed improvement in model performance (sensitivity analysis excluding smoking yielded an AUC of 0.841 [Figure S2], compared with 0.851 for the final model including smoking [Figure 3]) However, it is important to emphasize that our nomogram was developed using a 1:1 case‐control design, which artificially fixes the apparent prevalence at 50% and does not reflect the true prevalence of RA‐ILD in the general RA population. In real‐world settings, clinically significant ILD accounts for approximately 10‐18% of RA patients, with an additional 19‐67% having subclinical ILD, and the median survival of clinically diagnosed RA‐ILD is only 2‐8 years. Consequently, the predicted probabilities generated by our nomogram systematically overestimate absolute risk when applied to unselected clinical cohorts. The model should therefore be used for risk stratification and identification of high‐risk individuals rather than for estimating absolute disease probability. Recalibration of the model intercept using real‐world prevalence data would be necessary before the nomogram can be used for absolute risk estimation in clinical practice. Nevertheless, the model's discriminative ability (AUC = 0.851) remains valid for ranking individuals by relative risk, which is the primary intended use of this tool. Notably, the model includes readily available parameters‐‐‐age, MCHC, IL‐22, TNF‐α, and smoking‐‐‐that collectively outperform single biomarkers. Decision curve analysis confirmed its potential net benefit over default strategies across clinically relevant risk thresholds, suggesting utility in guiding early imaging or monitoring decisions.

Compared with previously published nomograms for RA‐ILD, our model offers several distinct contributions. First, it incorporates IL‐22 as a novel cytokine marker not previously included in RA‐ILD nomograms. Second, it focuses on a treatment‐naïve cohort, eliminating the confounding effects of immunosuppressive therapies on cytokine profiles. Third, it employs a comprehensive feature selection strategy combining both LASSO and Boruta algorithms. These features collectively enhance the model's utility for early identification of high‐risk patients.

Several limitations warrant consideration. First, the sample size was relatively small (n = 66), which may have limited the power to detect subtle differences (e.g., in IL‐17A) and increased the risk of overfitting despite cross‐validation. Although feature selection methods were employed to mitigate overfitting, the events‐per‐variable (EPV) ratio (EPV ≈ 6.6) remains below the commonly recommended threshold of 10, which may limit the robustness of the final model. Furthermore, the initial candidate pool subjected to feature selection included approximately twenty clinical and cytokine variables, making the effective overfitting pressure greater than the final EPV suggests. Second, the retrospective, single‐center design introduces potential selection bias and limits generalizability. Third, cytokine measurements were restricted to peripheral blood and may not fully reflect local lung microenvironment dynamics. Fourth, the nomogram should be regarded as exploratory rather than definitive, and its clinical applicability requires external validation in independent cohorts. Importantly, restricting the cohort to newly diagnosed, treatment‐naïve RA patients minimized the confounding effects of immunosuppressive therapies on cytokine levels, but may limit the generalizability of findings to patients receiving ongoing treatment. Additionally, inter‐observer reliability of the HRCT fibrosis score was not formally assessed, which may introduce measurement bias. Finally, no formal adjustment was applied for multiple comparisons across the nine cytokines tested, which may increase the risk of type I error; thus, our findings should be interpreted conservatively.

Future studies should focus on external validation of our nomogram in larger, prospective, multi‐center cohorts to confirm its generalizability. Longitudinal studies are also needed to evaluate whether baseline IL‐22 and TNF‐α levels can predict the future development of ILD over time. Additionally, incorporating established serological markers such as RF and anti‐CCP into the model, as well as exploring cytokine profiles in bronchoalveolar lavage fluid to better understand the local pulmonary immune environment, represent important directions for further investigation.

Furthermore, the absence of established serological markers such as rheumatoid factor and anti‐CCP antibodies represents another limitation, as these markers are well‐recognized correlates of RA‐ILD. Their omission from our model may weaken its clinical utility, and future studies should incorporate these markers to improve model performance.

In summary, our study provides novel insights into cytokine profiles associated with RA‐ILD in a treatment‐naïve cohort, offering a clearer view of underlying immune signatures unconfounded by immunosuppressive therapy. The identification of IL‐22 and TNF‐α as key correlates may inform future biomarker development, while the proposed nomogram offers a practical, hypothesis‐generating tool for clinicians to identify high‐risk patients who may benefit from HRCT screening. Although exploratory and requiring prospective validation before clinical implementation, this model represents a step toward personalized risk stratification in RA‐ILD.

## Author Contributions

**Li Lu:** writing – original draft. **Yongkang Chen:** data curation. **Ting Zhang:** resources. **Lina Cao:** resources. **Hua Ma:** data curation. **Yuanyuan Song:** software. **Dongmei Zhou:** project administration.

## Conflicts of Interest

The authors declare no conflicts of interest.

## Supporting information


Supporting File

## Data Availability

The datasets generated and/or analyzed during the current study are not publicly available due to patient privacy restrictions but are available from the corresponding author on reasonable request.
