# Supplementary material for: Analysis of Factors Associated With Interstitial Lung Disease in Treatment‐Naïve Rheumatoid Arthritis Patients and Development of a Classification Nomogram
Source: Immun Inflamm Dis. 2026 Oct 29;14(10):e70571. doi: 10.1002/iid3.70571 (PMC13720240; doi:10.1002/iid3.70571)
Supplement: Supplementary file 1 [file IID3-14-e70571-s001.docx]

**Supporting information**

**Table S1.** Adjusted comparisons of cytokine levels between RA‑ILD and RA‑non‑ILD groups, with adjustment for age and smoking history.

| **Cytokine** | **N** | **Beta (95% CI)** | **GMR (95% CI)** | ***P* value** |
| --- | --- | --- | --- | --- |
| IL_22 | 66 | -48.165 (-72.909, -23.422) |  | <0.001 |
| IL_17 | 66 | 0.064 (-0.541, 0.670) | 1.07 (0.58, 1.95) | 0.832 |
| IL_2 | 66 | 0.279 (-0.807, 1.364) | 1.32 (0.45, 3.91) | 0.610 |
| IL_6 | 66 | -0.129 (-0.854, 0.595) | 0.88 (0.43, 1.81) | 0.723 |
| IL_4 | 66 | 0.734 (-0.102, 1.571) | 2.08 (0.90, 4.81) | 0.084 |
| IL_10 | 66 | 0.208 (-0.368, 0.783) | 1.23 (0.69, 2.19) | 0.473 |
| IL_12P70 | 66 | 0.537 (-0.021, 1.095) | 1.71 (0.98, 2.99) | 0.059 |
| IFN_y | 66 | 0.621 (-0.227, 1.469) | 1.86 (0.80, 4.35) | 0.148 |
| TNF_a | 66 | 0.547 (0.044, 1.050) | 1.73 (1.05, 2.86) | 0.033 |

**Table S2.** Partial Spearman correlation analysis among cytokines and between cytokines and disease‑related clinical parameters, adjusting for age and smoking history.

| **Cytokine1** | **Cytokine2** | **Partial_Spearman_rho** | **P_value** | **P_FDR** | **n** | **Significant** | **FDR_significant** |
| --- | --- | --- | --- | --- | --- | --- | --- |
| IL_4 | IL_2 | 0.829421422 | 2.53266E-17 | 9.11756E-16 | 66 | P < 0.001 | Yes |
| IFN_y | IL_10 | 0.823463401 | 6.68143E-17 | 1.20266E-15 | 66 | P < 0.001 | Yes |
| IFN_y | IL_17 | 0.619513204 | 4.82044E-08 | 5.78453E-07 | 66 | P < 0.001 | Yes |
| TNF_a | IL_10 | 0.596982766 | 1.92246E-07 | 1.62195E-06 | 66 | P < 0.001 | Yes |
| IL_10 | IL_17 | 0.594285987 | 2.2527E-07 | 1.62195E-06 | 66 | P < 0.001 | Yes |
| IL_12P70 | IL_4 | 0.589083913 | 3.04612E-07 | 1.82767E-06 | 66 | P < 0.001 | Yes |
| IL_12P70 | IL_2 | 0.568676255 | 9.46991E-07 | 4.87024E-06 | 66 | P < 0.001 | Yes |
| TNF_a | IL_12P70 | 0.563431017 | 1.25219E-06 | 5.63487E-06 | 66 | P < 0.001 | Yes |
| TNF_a | IFN_y | 0.560927774 | 1.42837E-06 | 5.7135E-06 | 66 | P < 0.001 | Yes |
| TNF_a | IL_17 | 0.481928188 | 5.52835E-05 | 0.000199021 | 66 | P < 0.001 | Yes |
| IFN_y | IL_6 | 0.464981743 | 0.000108365 | 0.000354648 | 66 | P < 0.001 | Yes |
| TNF_a | IL_6 | 0.437756542 | 0.000297574 | 0.000892722 | 66 | P < 0.001 | Yes |
| IL_10 | IL_6 | 0.409020059 | 0.000791479 | 0.002191787 | 66 | P < 0.001 | Yes |
| TNF_a | IL_4 | 0.376317593 | 0.002178768 | 0.005602546 | 66 | P < 0.01 | Yes |
| IL_12P70 | IL_6 | 0.37241825 | 0.002441915 | 0.005860597 | 66 | P < 0.01 | Yes |
| IL_12P70 | IL_10 | 0.324699465 | 0.008851379 | 0.019915603 | 66 | P < 0.01 | Yes |
| IL_6 | IL_17 | 0.290566139 | 0.019847766 | 0.042030563 | 66 | P < 0.05 | Yes |
| IFN_y | IL_12P70 | 0.287024608 | 0.021473298 | 0.042946596 | 66 | P < 0.05 | Yes |
| IL_2 | IL_22 | 0.270354046 | 0.030725082 | 0.058215945 | 66 | P < 0.05 | No |
| IL_2 | IL_17 | -0.263952191 | 0.03507145 | 0.063128611 | 66 | P < 0.05 | No |
| IFN_y | IL_2 | -0.252345901 | 0.044255466 | 0.075866513 | 66 | P < 0.05 | No |
| IL_6 | IL_4 | 0.225752181 | 0.07286822 | 0.119238906 | 66 | NS | No |
| IL_4 | IL_17 | -0.184234711 | 0.145023622 | 0.226993495 | 66 | NS | No |
| IL_17 | IL_22 | -0.178793052 | 0.157487492 | 0.236231239 | 66 | NS | No |
| TNF_a | IL_2 | 0.169014689 | 0.181844905 | 0.261856663 | 66 | NS | No |
| TNF_a | IL_22 | -0.143438029 | 0.258161714 | 0.357454681 | 66 | NS | No |
| IL_12P70 | IL_17 | 0.126031675 | 0.32102902 | 0.424476283 | 66 | NS | No |
| IL_6 | IL_2 | 0.122481598 | 0.33495708 | 0.424476283 | 66 | NS | No |
| IL_12P70 | IL_22 | 0.120736883 | 0.341939228 | 0.424476283 | 66 | NS | No |
| IL_4 | IL_22 | 0.111250037 | 0.381475868 | 0.457771042 | 66 | NS | No |
| IL_10 | IL_2 | -0.098587926 | 0.438310361 | 0.509005581 | 66 | NS | No |
| IFN_y | IL_4 | -0.075399991 | 0.553746623 | 0.622964951 | 66 | NS | No |
| IL_6 | IL_22 | 0.07174168 | 0.573197677 | 0.625306557 | 66 | NS | No |
| IFN_y | IL_22 | -0.055118484 | 0.665315377 | 0.704451576 | 66 | NS | No |
| IL_10 | IL_4 | 0.028818241 | 0.821163335 | 0.844625145 | 66 | NS | No |
| IL_10 | IL_22 | -0.021816555 | 0.864135023 | 0.864135023 | 66 | NS | No |

**Figure S1.** Comparison of plasma IL-22 levels between RA-ILD and RA-non-ILD groups. Data are presented as mean ± SD. *P* < 0.001 (independent samples t‑test).

Figure S2. Cross‑validated ROC curve of the four‑variable model (age, MCHC, IL‑22, and ln_TNF‑α) without smoking, with an AUC of 0.841 (95% CI: 0.744-0.939). This sensitivity analysis is provided for comparison with the final model including smoking (Figure 3).
